# A Theoretical and Experimental Study of a Piezoelectric Pump with Two Elastic Chambers

**DOI:** 10.3390/s20205867

**Published:** 2020-10-16

**Authors:** Xiaolong Zhao, Dingxuan Zhao, Qinghe Guo

**Affiliations:** School of Mechanical Engineering, Yanshan University, Qinhuangdao 066004, China; zxlysu@139.com (X.Z.); qinghekwok@126.com (Q.G.)

**Keywords:** piezoelectric pump, elastic chamber, dynamic load, elastic diaphragm

## Abstract

The paper is a continuation of our work on the dynamic load in piezoelectric pumps. In the study, the dynamic load of liquid in the pipelines was proposed as a key factor that limits the output performance of piezoelectric pumps. To decrease the dynamic load, a piezoelectric pump with two elastic chambers was proposed in our previous published work. In this paper, the performance and key parameters of the piezoelectric pump with two elastic chambers were studied through theoretical analyses and experimental tests. After establishing the mathematical model of the piezoelectric pump with two elastic chambers, the paper theoretically analyzed the performance of the pump and the effect of different structural parameters on the performance. Then prototypes with a range of structural parameters were developed and tested. As revealed from the test results, the elastic chamber effectively decreased the dynamic load of the liquid in the pipelines and the flow rate of the prototype with two elastic chambers was higher than that of the prototype with one or no elastic chamber. However, the elastic chamber did not lead to the increase in the maximum output backpressure of the prototype. Adopting an elastic diaphragm exhibiting a smaller stiffness or a larger diameter could help decrease the dynamic load of the liquid. The elastic chamber more significantly impacted the flow rate of the piezoelectric pump with long pipelines. The pump chamber height had a significant effect on the output performance of the piezoelectric pump with two elastic chambers, which is consistent with the conventional piezoelectric pump. At the height of 0.2 mm, the flow rate of the prototype with two elastic chambers was peaked at 7.7 mL/min; at the height of 0.05 mm, the output backpressure reached the highest of 28.2 kPa. The dynamic load could decrease the amplitude of the piezoelectric vibrator, whereas the prototype with two elastic chambers could effectively reduce the impact of dynamic load on the piezoelectric vibrator. The flow rate decreased almost linearly with the backpressure. Under the same backpressure, the flow rate of the prototype with two elastic chambers was higher than that of the prototype without elastic chamber, and the flow rate difference between the two prototypes gradually decreased with the backpressure.

## 1. Introduction

Piezoelectric pumps can be used in many fields (e.g., medicine, MEMS and fuel cells) for their advantages of simple structure, high actuation strength, easy miniaturization, no magnetic influence and low noise [1,2,3,4,5,6,7,8,9]. In recent years, with the continuous expansion of the application field of piezoelectric pumps, many new application fields that require piezoelectric pumps to have higher output performance have appeared. Unfortunately, the existing output performance of piezoelectric pumps cannot fully satisfy the application requirements in many emerging fields. Therefore, the output performance of piezoelectric pumps should be urgently improved. Improving the output performance of piezoelectric pumps is a very complex and difficult challenge, especially without increasing the volume and input power of the pump. It is worth noting that increasing the operating frequency is one of the most simple and convenient potential ways to improve the performance of piezoelectric pumps without increasing the volume and input power of the pumps.

However, the high driving frequency will cause considerable problems, seriously decreasing the output performance and efficiency of piezoelectric pump. These considerable problems mainly focus on the following three aspects. The first aspect is that the hysteresis of the check valve at high frequency seriously reduces the output flow of piezoelectric pumps. The hysteresis means that the vibration of check valves lags behind that of the piezoelectric actuator [10]. Zhang et al. studied the hysteresis of check valves and found that the hysteresis caused the flow rate of piezoelectric pumps to decrease rapidly at high driving frequency [11]. Since there is a time delay when the fluid in the pump chamber transmits the vibrational mechanical energy, the opening and closing of passive check valves lags behind the vibration of the piezoelectric actuator. The hysteresis of the valves was not obvious at low driving frequency, but it was significant at high driving frequency. Obviously, when the piezoelectric pump sucks liquid, the hysteresis of opening the inlet valve reduces the amount of liquid sucked into the pump chamber, and the hysteresis of closing the outlet valve causes the liquid in the outlet pipe to flow back into the pump chamber. When the pump discharges liquid, the hysteresis of opening the outlet valve reduces the amount of liquid discharged from the pump chamber, and the hysteresis of closing the inlet valve makes the liquid in the pump chamber return to the inlet pipe again. Researchers have been committed to designing valves with high-frequency response to solve this problem [12,13,14,15,16]. The existing research in this field has become mature, and the response frequency of some high-frequency valves reachs over 1 kHz. The second aspect is that piezoelectric pumps are prone to severe cavitation at high driving frequency. Zhang et al. [17] studied the cavitation phenomenon of piezoelectric pumps and found that the cavitation phenomenon became more serious as the driving frequency increased. When the piezoelectric pump sucks a liquid, the volume of the pump chamber becomes larger and the pressure in the chamber decreases, so the gas dissolved in the liquid escapes and produces cavitation. He et al. [18] studied the harm of cavitation to piezoelectric pumps. Cavitation leads to a decrease in the bulk modulus of the working fluid in the piezoelectric pump, thereby severely reducing the efficiency of the pump. To inhibit cavitation, Kim Gie downregulated the amount of gas in the fluid by presetting the pressure on the fluid [19]. As suggested from the experimental results, this method is feasible. The third aspect is that some local structures of the piezoelectric pump weaken the high-frequency output performance. For example, the structure and volume of the pump chamber, the structure of the valve hole, and the diameter of the pipeline all have an important influence on the high-frequency output performance of the piezoelectric pump [20,21,22]. The problem of structure weakening high-frequency performance is particularly prominent in valveless piezoelectric pumps. The valveless piezoelectric pump eliminates the effect of the check valve on performance, so it is suitable for working at high driving frequency. However, some local structures of the pump reduce its high-frequency output performance. For example, valveless piezoelectric pumps have poor cut-off properties of inlet and outlet of pump chamber, resulting in low output flow rate and pressure at high frequency. To improve the high-frequency output performance of valveless piezoelectric pumps, some scholars optimized the structure of pipelines and pump chamber. For example, scholars proposed a valveless piezoelectric pump with Cone-shaped tubes [23], a valveless piezoelectric pump with Y-shape tubes [24] and a valveless piezoelectric pump with unsymmetrical slope chamber bottom [25]. The optimization of the local structure of the valveless piezoelectric pump greatly improves the high-frequency output performance.

The mentioned studies have noticeably contributed to enhancing effective frequency of the pump, and the output performance of the pump has been effectively enhanced. Therefore, studying various problems caused by high driving frequency is of great significance to improve the working frequency and the pump performance.

The reciprocating motion of piezoelectric vibrator results in high frequency vibration of liquid in the pump system [26]. The vibration of the liquid causes its velocity pulsation phenomenon. Moreover, the vibration of the liquid will generate inertial force. The inertial force is regarded as a dynamic load that increases the power loss of the pump system. In this study, the dynamic load of the liquid in the inlet and outlet pipelines is proposed as one of the vital reasons for reducing the output performance of piezoelectric pumps. Since the liquid in the pump system is primarily distributed in the inlet and outlet pipelines, the dynamic load of the liquid in the pipelines largely causes the performance of piezoelectric pumps to decrease at high frequency. To decline the dynamic load of liquid, a piezoelectric pump with two elastic chambers was proposed in the present study. The elastic chamber in this pump is capable of cutting off the rigid connection between the liquid in the pump chamber and the liquid in the pipelines. Thus, the elastic chamber is similar to accumulator in fluid transmission system, so the vibration of the liquid can be effectively reduced. Thus, the velocity pulsation is smoothed, and the dynamic load of the liquid in the pipelines decreases. Obviously, this study uses a method different from those mentioned above to improve the performance of piezoelectric pumps.

We have conducted continuous research on the dynamic load of piezoelectric pumps. In our previous study [27], we had preliminarily verified that the elastic chamber can reduce the dynamic load. In that paper we proposed a piezoelectric pump with two elastic chambers and studied the effectiveness of the elastic chamber in reducing dynamic load with the needle valve fully open and 50% needle valve opening. In addition, the effect of the elastic chamber height on its function was also studied. In the present paper we focus on the effect of different structural parameters on the performance of a piezoelectric pump with two elastic chambers. The performance of the piezoelectric pump with two elastic chambers was studied by theoretical analyses and experimental tests. Firstly, a mathematical model of a piezoelectric pump with two elastic chambers was established. Based on this model, the performance of the pump and the effect of different structural parameters on the performance were analyzed theoretically. Then we developed prototypes with a range of structural parameters and conducted tests on these prototypes.

## 2. Theoretical Analysis

**Figure 1:**
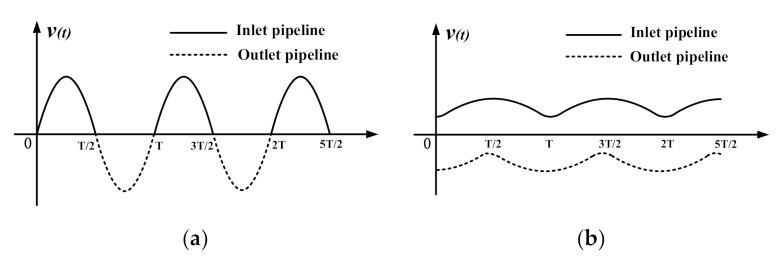
Vibration velocity curves of liquid in the inlet and outlet pipelines. (**a**) The piezoelectric pump without the elastic chamber; (**b**) the piezoelectric pump with the elastic chambers.

To introduce the design concept of the piezoelectric pump with two elastic chambers, the concept of dynamic load should be explained first. According to the structure and working principle of piezoelectric pumps, the piezoelectric pump system can be considered a vibration system and can be simplified as a vibration model in which a piezoelectric actuator drives the liquid to conduct simple harmonic vibration. The inlet and outlet check valves make the liquid in the inlet and outlet pipelines move in one direction, as shown in Figure 1a. In the present study, the inertial force of the liquid during vibration is termed the dynamic load. The dynamic load refers to an additional load on the piezoelectric actuator, causing an energy loss and reducing the output performance of the piezoelectric pump. Since the liquid mass in the pipelines is larger than the liquid mass in the pump chamber, the dynamic load of the liquid in the pipelines is larger than that of the liquid in the pump chamber. Accordingly, the dynamic load of the liquid in the pipelines primarily restricts the further enhancement of the output performance of the piezoelectric pump.

The vibration theory of mechanical system is followed to analyze the generation mechanism of the dynamic load. When the piezoelectric vibrator is driven by a sinusoidal signal, and the amplitude of the vibrator is assumed as $\Phi_{1}$, the vibration displacement $X_{1}$ of the liquid in the pump chamber is defined as [27]:

(1) $$X_{1}=\Phi_{1}\sin(2\pi ft)$$

The liquid in the pump system is assumed to be incompressible. When the pump is working, the liquid in the pump chamber and the liquid in the outlet pipeline are regarded as rigid connections, so the vibration displacement $X_{2}$ of the liquid in the inlet pipeline is:

(2) $$X_{2}=\left\{\begin{array}{cc} L_{2}\sin(2\pi ft) & nT\leq t\leq (n+\frac{1}{2})T\\ 0 & (n+\frac{1}{2})T\leq t\leq (n+1)T \end{array}\right.$$

In Equation (2), *L_2_* denotes the amplitude of the liquid in the inlet pipeline. If it is assumed that there is no leakage in the pump chamber, all the liquid sucked from the inlet pipeline flows into the pump chamber within each suction cycle. Assume that the cross-sectional areas of the pump chamber and inlet pipeline are $A_{1}$ and $A_{2}$, respectively. Thus, $X_{1}$ and $X_{2}$ satisfy the following equation:

(3) $$A_{1}\overset{•}{X_{1}}\rho =A_{2}\overset{•}{X_{2}}\rho$$

In Equation (3), ρ is liquid density. Equation (4) can be obtained by substituting Equation (1) and Equation (2) into Equation (3):

(4) $$L_{2}=\left\{\begin{array}{cc} \frac{A_{1}\Phi_{1}}{A_{2}} & nT\leq t\leq (n+\frac{1}{2})T\\ 0 & (n+\frac{1}{2})T\leq t\leq (n+1)T \end{array}\right.$$

Assuming that the mass of the liquid in the inlet pipeline is $m_{2}$, $m_{2}$ satisfies the following equation:

(5) $$m_{2}=A_{2}l_{2}\rho$$

In Equation (5), $l_{2}$ is the length of the inlet pipeline. According to Newton’s second law, the dynamic load $F_{2}$ of the liquid in the inlet pipeline is:

(6) $$F_{2}=\left\{\begin{array}{cc} m_{2}\overset{••}{X_{2}} & nT\leq t\leq (n+\frac{1}{2})T\\ 0 & (n+\frac{1}{2})T\leq t\leq (n+1)T \end{array}\right.$$

Next, Equation (7) can be obtained by substituting Equations (2), (4) and (5) into Equation (6):

(7) $$F_{2}=\left\{\begin{array}{cc} A_{1}l_{2}\rho \Phi_{1}{\left(2\pi f\right)}^{2}\sin(2\pi ft) & nT\leq t\leq (n+\frac{1}{2})T\\ 0 & (n+\frac{1}{2})T\leq t\leq (n+1)T \end{array}\right.$$

Using a similar derivation process, we can obtain the dynamic load $F_{3}$ of the liquid in the outlet pipeline:

(8) $$F_{3}=\left\{\begin{array}{cc} 0 & nT\leq t\leq (n+\frac{1}{2})T\\ A_{1}l_{3}\rho \Phi_{1}{\left(2\pi f\right)}^{2}\sin(2\pi ft) & (n+\frac{1}{2})T\leq t\leq (n+1)T \end{array}\right.$$

In Equation (8), $l_{3}$ is the length of the outlet pipeline, so the resultant force of $F_{2}$ and $F_{3}$ acting on the piezoelectric vibrator is:

(9) $$F_{1}=\left\{\begin{array}{cc} A_{1}l_{2}\rho \Phi_{1}{\left(2\pi f\right)}^{2}\sin(2\pi ft) & nT\leq t\leq (n+\frac{1}{2})T\\ A_{1}l_{3}\rho \Phi_{1}{\left(2\pi f\right)}^{2}\sin(2\pi ft) & (n+\frac{1}{2})T\leq t\leq (n+1)T \end{array}\right.$$

Obviously, the amplitude of $F_{1}$ is proportional to the square of the driving frequency f, and proportional to the length of the pipelines and vibrator amplitude $\Phi_{1}$. Accordingly, the dynamic load increases quadratically with the driving frequency f.

The dynamic load of liquid in the pipelines results from the liquid vibration, so reducing the liquid vibration in the pipelines helps decrease the dynamic load. To decrease the dynamic load in the inlet and outlet pipelines, a piezoelectric pump with two elastic chambers was designed. In brief, the design concept of the piezoelectric pump with two elastic chambers is that an elastic chamber is respectively built outside the inlet and outlet valves to decrease the dynamic load in the pipelines. The elastic chamber can reduce the liquid vibration in the inlet and outlet pipelines and smooth the output flow rate, as shown in Figure 1b.

**Figure 2:**
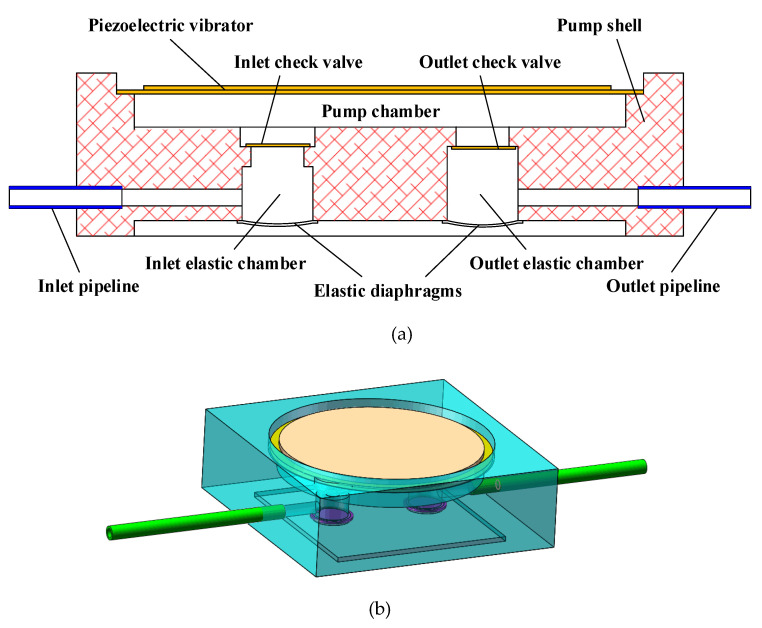
The structure diagram of the pump with two elastic chambers. (**a**) Two-dimensional structure; (**b**) three-dimensional structure.

The structure of piezoelectric pump with two elastic chambers is illustrated in Figure 2. The two elastic chambers consist of an inlet elastic chamber and an outlet elastic chamber, respectively located below the inlet check valve and the outlet check valve. The elastic chamber is composed of a chamber below the check valve and an elastic diaphragm.

**Figure 3:**
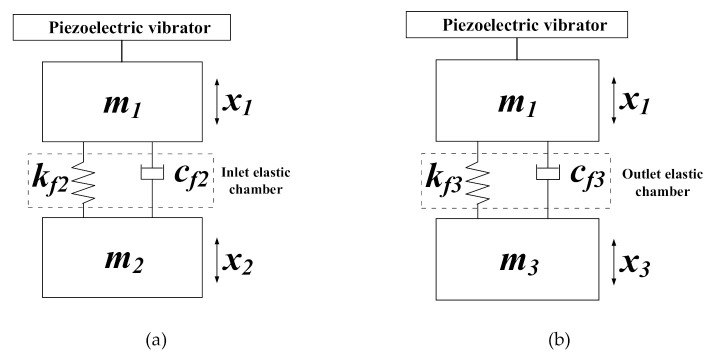
The simplified vibration model of the pump with two elastic chambers. (**a**) The simplified model with the inlet valve open; (**b**) the simplified model with the outlet valve open.

When the pump with two elastic chambers works, the inlet and outlet elastic chambers block the rigid connection between the liquid in the pump chamber and the liquid in the pipelines. The flow resistance caused by the inlet and outlet check valves is ignored. Subsequently, the simplified vibration model of the pump system can be built, as illustrated in Figure 3. In the simplified vibration model, $m_{1}$ denotes the liquid mass in the pump chamber, $m_{2}$ represents the liquid mass in the inlet pipeline and $m_{3}$ represents the liquid mass in the outlet pipeline. Elastic element $k_{f2}$ and damping element *c_f2_* represent the function of the inlet elastic chamber. Likewise, elastic element $k_{f3}$ and damping element $c_{f3}$ represent the function of the outlet elastic chamber. $x_{1}$ represents the vibration displacement of the liquid in the pump chamber. $x_{2}$ and $x_{3}$ respectively represent the vibration displacement of the liquid in the inlet and outlet pipelines.

According to D’Alembert’s principle, a force analysis was conducted on the liquid mass $m_{2}$, and the motion differential equation of the liquid in the inlet pipeline was built, as expressed in Equation (10). For a detailed derivation of Equation (10), please refer to our previous article [27]:

(10) $$m_{2}\overset{••}{x_{2}}+c_{f2}\overset{•}{x_{2}}+k_{f2}x_{2}=k_{f2}\Phi_{1}\sin(2\pi ft)+c_{f2}\Phi_{1}(2\pi f)\cos(2\pi ft)$$

The vibration displacement $x_{2}$ can be obtained from Equation (10):

(11) $$x_{2}=\Phi_{1}H_{2}\sin(2\pi ft-\varphi_{2})$$

where:

(12) $$H_{2}=\sqrt{\frac{1+{\left(2\zeta_{2}\lambda_{2}\right)}^{2}}{{\left(1-\lambda_{2}{}^{2}\right)}^{2}+{\left(2\zeta_{2}\lambda_{2}\right)}^{2}}}$$

(13) $$\varphi_{2}=tg^{-1}\frac{2\zeta_{2}\lambda_{2}{}^{3}}{1-\lambda_{2}{}^{2}+{\left(2\zeta_{2}\lambda_{2}\right)}^{2}}$$

(14) $$\lambda_{2}=\frac{\omega }{\omega_{n2}}=\frac{f}{f_{n2}}$$

(15) $$\zeta_{2}=\frac{c_{f2}}{2m_{2}}$$

(16) $$f_{n2}=\frac{1}{2\pi }\omega_{n2}$$

*H*_2_ denotes the amplitude amplification factor, $\lambda_{2}$ denotes the frequency ratio, $\varphi_{2}$ denotes phase angle, and $\zeta_{2}$ denotes the damping ratio. $f_{n2}$ is the natural frequency of the vibration model (Figure 3a). $\omega_{n2}$ is the natural angular frequency corresponding to $f_{n2}$.

According to Newton’s second law and Equation (11), we can obtain the new dynamic load $\bar{F_{2}}$ of the liquid in the inlet pipeline, as shown in Equation (17):

(17) $$\bar{F_{2}}=m_{2}\overset{••}{x_{2}}=A_{2}l_{2}\rho \Phi_{1}H_{2}{\left(2\pi f\right)}^{2}\sin(2\pi ft-\varphi_{2})$$

Likewise, the new dynamic load $\bar{F_{3}}$ of the liquid in the outlet pipeline is:

(18) $$\bar{F_{3}}=m_{3}\overset{••}{x_{3}}=A_{3}l_{3}\rho \Phi_{1}H_{3}{\left(2\pi f\right)}^{2}\sin(2\pi ft-\varphi_{3})$$

The resultant force of $\bar{F_{2}}$ and $\bar{F_{3}}$ acting on the piezoelectric vibrator is a new dynamic load $\bar{F_{1}}$. So the new dynamic load $\bar{F_{1}}$ of the piezoelectric pump with the inlet and outlet elastic chambers is:

(19) $$\bar{F_{1}}=\left\{\begin{array}{cc} A_{2}l_{2}\rho \Phi_{1}H_{2}{\left(2\pi f\right)}^{2}\sin(2\pi ft-\varphi_{2}) & nT\leq t\leq (n+\frac{1}{2})T\\ A_{3}l_{3}\rho \Phi_{1}H_{3}{\left(2\pi f\right)}^{2}\sin(2\pi ft-\varphi_{3}) & (n+\frac{1}{2})T\leq t\leq (n+1)T \end{array}\right.$$

After comparing $\bar{F_{1}}$ and *F_1_*, it can be found that $\bar{F_{1}}$ is less than *F_1_*. So the dynamic loads in the inlet and outlet pipelines and their resultant force on the piezoelectric vibrator are both reduced.

In addition, the dynamic loads of the piezoelectric pump with the inlet elastic chamber and the piezoelectric pump with the outlet elastic chamber are also analyzed. According to Equations (9) and (19), we can easily obtain the dynamic load $\bar{F_{11}}$ of the piezoelectric pump with the inlet elastic chamber and the dynamic load $\bar{F_{12}}$ of the piezoelectric pump with the outlet elastic chamber:

(20) $$\bar{F_{11}}=\left\{\begin{array}{cc} A_{2}l_{2}\rho \Phi_{1}H_{2}{\left(2\pi f\right)}^{2}\sin(2\pi ft-\varphi_{2}) & nT\leq t\leq (n+\frac{1}{2})T\\ A_{3}l_{3}\rho \Phi_{1}{\left(2\pi f\right)}^{2}\sin(2\pi ft) & (n+\frac{1}{2})T\leq t\leq (n+1)T \end{array}\right.$$

(21) $$\bar{F_{12}}=\left\{\begin{array}{cc} A_{1}l_{2}\rho \Phi_{1}{\left(2\pi f\right)}^{2}\sin(2\pi ft) & nT\leq t\leq (n+\frac{1}{2})T\\ A_{3}l_{3}\rho \Phi_{1}H_{3}{\left(2\pi f\right)}^{2}\sin(2\pi ft-\varphi_{3}) & (n+\frac{1}{2})T\leq t\leq (n+1)T \end{array}\right.$$

Assuming that the structural parameters of the inlet and outlet pipelines are the same, *A_2_* is equal to *A_3_*, and *l_2_* is equal to *l_3_*. Assuming that the structural parameters of the inlet and outlet elastic chambers are the same, *H_2_* is equal to *H_3_*, and $\varphi_{2}$ is equal to $\varphi_{3}$. After analyzing Equations (20) and (21), it can be found that the power loss caused by $\bar{F_{11}}$ is equal to the power loss caused by $\bar{F_{12}}$.

High driving frequency *f* makes the frequency ratio far greater than 1, thus making *H_2_* and *H_3_* respectively satisfy $H_{2}\approx \frac{1}{\lambda_{2}^{2}}$ and $H_{3}\approx \frac{1}{\lambda_{3}^{2}}$. So Equation (19) can be modified to:

(22) $$\bar{F_{1}}\approx \left\{\begin{array}{cc} A_{2}l_{2}\rho \Phi_{1}\frac{1}{\lambda_{2}^{2}}{\left(2\pi f\right)}^{2}\sin(2\pi ft-\varphi_{2})=k_{f2}\Phi_{1}\sin(2\pi ft-\varphi_{2}) & nT\leq t\leq (n+\frac{1}{2})T\\ A_{3}l_{3}\rho \Phi_{1}\frac{1}{\lambda_{3}^{2}}{\left(2\pi f\right)}^{2}\sin(2\pi ft-\varphi_{3})=k_{f3}\Phi_{1}\sin(2\pi ft-\varphi_{3}) & (n+\frac{1}{2})T\leq t\leq (n+1)T \end{array}\right.$$

Equation (22) shows that the elastic chamber almost eliminates the influence of driving frequency *f* and the liquid mass on the dynamic load. When the piezoelectric pump has high driving frequency *f* and small elastic stiffness (*k_f2_* and *k_f3_*), we can obtain $\bar{F_{1}}<<F_{1}$. So the mechanism analysis indicates that the dynamic load is effectively decreased by the elastic chamber.

Finally, the effect of the pump chamber height on the output performance of the piezoelectric pump is analyzed. Assuming that the initial volume of the pump chamber is *V_0_*, and the volume change of the pump chamber in one cycle is ΔV. Based on the definition of liquid bulk modulus in fluid mechanics, when the volume of the pump chamber is compressed, the pressure *P* in the pump chamber is:

(23) $$P=\beta_{e}\frac{2\Delta V}{2V_{0}+\Delta V}$$

In Equation (23), *β_e_* is the equivalent bulk modulus of the liquid in the pump chamber. Under the condition of keeping other structural parameters of the pump chamber unchanged, reducing the height of the pump chamber will reduce the initial volume *V_0_* of the pump chamber. According to Equation (23), reducing the initial volume *V_0_* will increase the output pressure *P* of pump chamber, so reducing the height of the pump chamber helps to increase the output pressure *P* of pump chamber.

When the height of the pump chamber is small, the liquid flow in the pump chamber can be analyzed by using the model of gap flow in flat plate, so the pressure loss ΔP in the pump chamber is:

(24) $$\Delta P=\frac{12\mu l}{h^{2}}v$$

In Equation (24), *μ* is the fluid kinematic viscosity coefficient, *l* is the length of the gap, *h* is the height of the gap, and *v* is the average flow rate of the fluid. Equation (24) shows that the pressure loss ΔP is inversely proportional to the square of height *h*. The height *h* here represents the height of the pump chamber. Therefore, if the pump chamber height is too small, the output performance of the pump will be seriously reduced. In total, the output performance of piezoelectric pump can be improved by properly reducing the height of pump chamber, but the output performance will be reduced if the chamber height is too low.

## 3. Prototype and Experimental Device

### 3.1. Prototype Parameters

**Figure 4:**
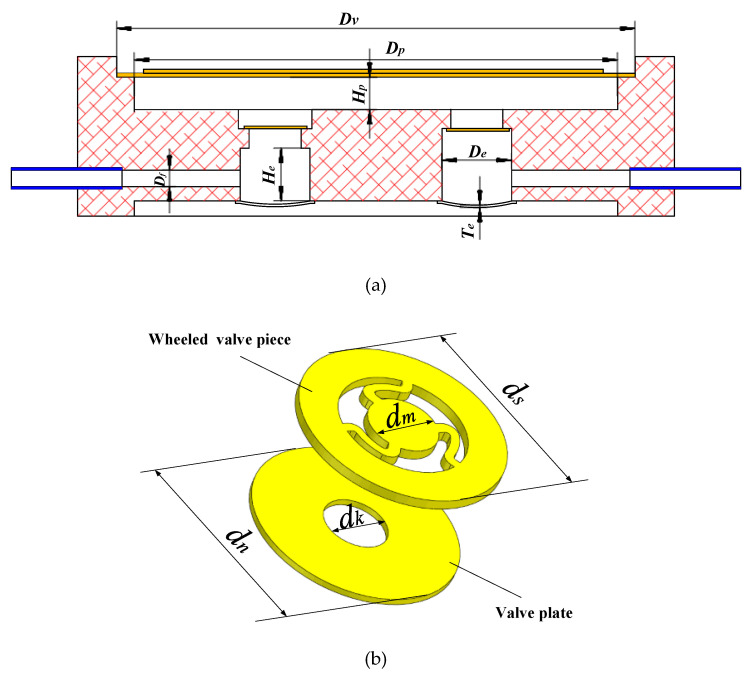
Key structural parameters of the piezoelectric pump with two elastic chambers. (**a**) Main structure of the pump. (**b**) Structure of the wheel valve.

Figure 4a shows the key structural parameters of the piezoelectric pump with two elastic chambers. *D_v_* is the diameter of the piezoelectric vibrator, *D_p_* is the diameter of the pump chamber and *H_p_* is the height of the pump chamber. *D_f_* is the diameter of the inlet and outlet flow channel. *T_e_* is the thickness of the elastic diaphragm, *H_e_* is the height of the elastic chamber and *D_e_* is the diameter of the elastic diaphragm. Figure 4b shows the key structural parameters of the wheel check valve. The check valve is formed by bonding the valve piece and the valve plate. *d_s_* and *d_m_* respectively denote the diameter of the fixed part and moving part of the valve piece. *d_n_* and *d_k_* respectively denote the diameter of the outer substrate and center hole of the valve plate. *k_v_* indicates the stiffness of the valve in the opening direction. The main structural parameters of the test prototype with two elastic chambers are shown in Table 1.

**Figure 5:**
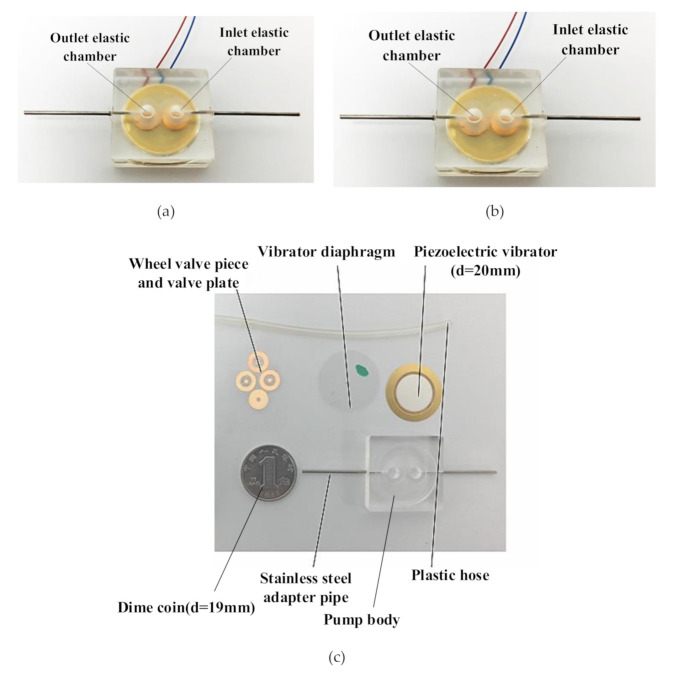
Photo of the fabricated piezoelectric pump with two elastic chambers; (**a**) top view; (**b**) bottom view; (**c**) the main components of the prototype.

**Figure 6:**
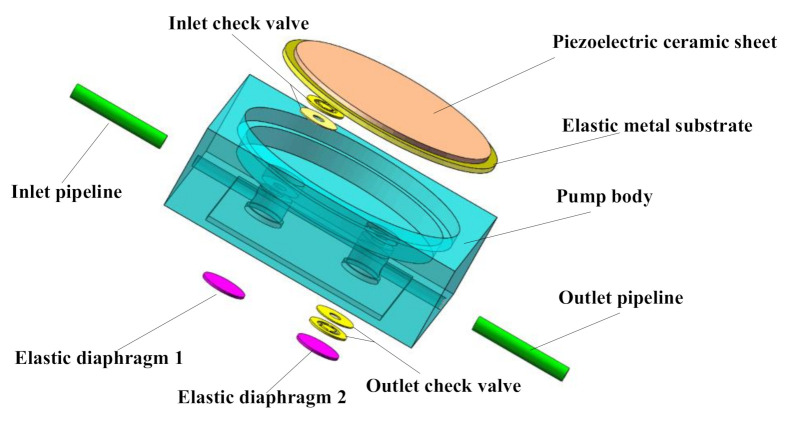
Exploded view of the designed micropump.

Figure 5 and Figure 6 respectively illustrate a photo and an exploded view of the piezoelectric pump designed in this study. Its overall dimensions were 25 mm × 25 mm × 10 mm. A dime coin of RMB (Φ=19 mm) was used as a size reference, as shown in Figure 5c. The pump body was made of polymethylmethacrylate (PMMA), a transparent PMMA material that is easy to cut. In addition, the material has high light transmittance, facilitating the curing of UV light solid glue. As shown in Figure 5c, the pump body is a monolithic structure that was machined by a precision engraving machine, and the precision engraving machine is a CNC engraving and milling machine, which is smaller in size than the CNC and excels at milling small precision parts. A JD80V CNC engraving and milling machine (BEIJING JINGDIAO, Beijing City, China) was used. The type and size of the cutter head can be chosen according to the need, but most of the diameter is between 1 mm to 5 mm. Silica gel diaphragms exhibit high elasticity and strong corrosion resistance. Thus, a silica gel diaphragm was selected to make the elastic diaphragm. The term silica gel diaphragm refers to a common industrial product, and silica gel diaphragms (HD-4452) with different thicknesses were purchased to make an elastic diaphragm. The elastic diaphragm was bonded to the bottom of the pump body’s elastic chamber with UV solid glue, which cures quickly when bonding parts, and it is widely used to bond and secure electronic components. The brand of glue we use is Valigoo (LELAI, Dongguan, China) model number V-3218. Beryllium bronze is characterized by high strength, hardness, elastic and fatigue limits, as well as low elastic hysteresis, so beryllium bronze sheet (C17200) was chosen to make the wheel check valves, which are made by UV laser cutting and tested by a stiffness test bench of 355.31 N/m. First, we bonded the valve plate and the valve plate with epoxy adhesive and then, we bonded the valve plate to the valve hole seat of the pump body with UV solid glue. We used polyester adhesive to bond the piezoelectric ceramic sheet to a flexible metal substrate to make the vibrator, polyester tape was used as a waterproof vibrating membrane fixed to the bottom of the metal substrate, and the piezoelectric vibrator was fixed to the top of the pump chamber of the pump body with UV solid glue.

### 3.2. Experimental System

**Figure 7:**
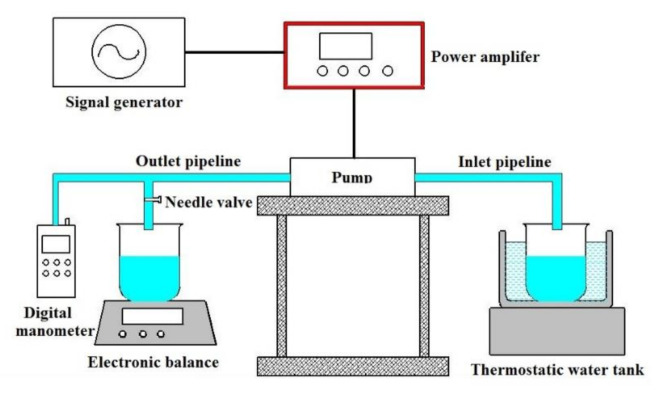
Schematic diagram of the performance test system.

A performance test system was developed (Figure 7). We used asignal generator (DG 1022 Rigol, Beijing, China) to generate a sinusoidal driving signal with a 0°phase shift. Then the sinusoidal driving signal was amplified by a power amplifier (HH-2, Yilong, Changzhou, China) to actuate the piezoelectric vibrator. To adjust the back pressure of the system, we installed a needle valve at the end of the outlet pipe. To keep the back pressure of the pump with needle valve fully open at 0, we placed the pipelines horizontally on the test bench. We used an electronic balance to measure the weight change of the water tank, thereby indirectly calculating the output flow of the piezoelectric pump. We installed a digital manometer on the outlet pipeline to measure the output back pressure. To ensure the accuracy of measurement data, each prototype was measured four times and the average value was calculated.

Bubbles in the water will seriously reduce the output stability of the pump and reduce the accuracy of the test. To eliminate air bubbles in the water, we used a thermostatic water bath to keep the water temperature at 60 °C during the testing process. Next, we further explain why the water temperature was kept at 60 °C. The effect of temperature on the performance of piezoelectric pump had been studied in detail [28]. In the published article, as the temperature of the measured liquid gradually increases from 30 °C to 80 °C, the output flow of the piezoelectric pump also increases. In addition, it is observed that as the temperature increases, the content of bubbles in the flow channels and pump cavity gradually decreases. The adverse effects of bubbles on piezoelectric pumps have been widely studied. For example, bubbles seriously reduce the stability of the output flow and pressure of the piezoelectric pump. Therefore, in order to reduce the bubble content and improve the stability of the output performance of the tested pumps, we heated the water in the test and kept it at 60 °C. Considering that we only study the effect of the elastic chamber on the performance of the pump, and do not study the effect of temperature on the performance, we keep the constant water temperature in the entire test. To reduce the interference of bubbles, we keep the water temperature at 60 °C. Of course, the water temperature can also be kept at other temperatures.

## 4. Results and Analysis

### 4.1. Performance Comparison of Prototypes with Different Numbers of Elastic Chamber

To ascertain the performance improvement of the piezoelectric pump by the elastic chamber, a prototype with an inlet elastic chamber, the prototype with an outlet elastic chamber and the prototype without elastic chamber were also developed as comparative test prototypes. For the prototype with two elastic chambers, the height of pump chamber was 0.15 mm, the stiffness of wheeled check valves was 355.31 N/m, the thickness of elastic diaphragms was 0.6 mm, and the diameter of elastic diaphragms was 5 mm. The three mentioned comparative test prototypes exhibited the identical main structural parameters to the prototype with two elastic chambers. All prototypes used identical inlet and outlet pipelines with a diameter of 1.5 mm and a length of 140 mm. The inlet and outlet elastic chambers employed identical elastic diaphragms with a thickness of 0.6 mm and a diameter of 5 mm. We set the voltage amplitude of sinusoidal drive signal to 150 Vpp. In the range of 10 Hz–290 Hz, we take a frequency as the driving frequency at every 20 Hz.

**Figure 8:**
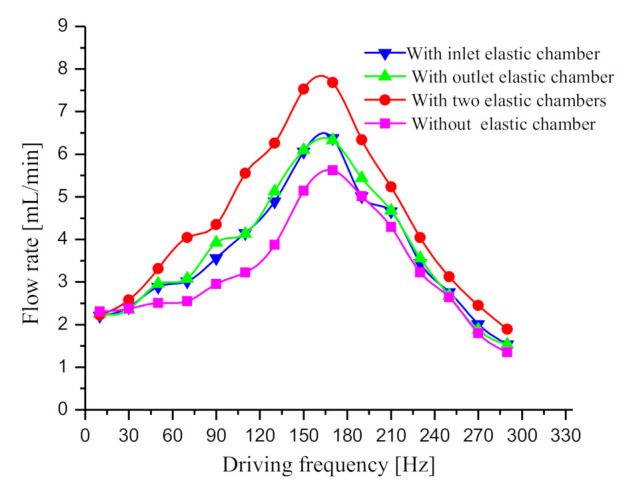
Flow rate curves of the four prototypes.

After opening the needle valve, the driving frequency was altered in turn, and the flow rates of the four prototypes were measured. The test result of flow rate is presented in Figure 8; with the increase in the driving frequency, the flow rate curves of the four prototypes generally increase first and then decline. As revealed from the test result (Figure 8), the inlet elastic chamber and the outlet elastic chamber effectively reduced the dynamic load, which helps improve the flow rate of the piezoelectric pump. The maximum output flow rate of the prototype with two elastic chambers is nearly 36% higher than that of the prototype without elastic chamber. Based on the analysis of Equations (9) and (19)–(21) in Section 2, the flow rate curves are analyzed and explained. First, at the low driving frequency (*f* < 30 Hz), the four prototypes exhibit low flow rate differences. The frequency ratio *λ* at low driving frequency is relatively low, so the elastic chamber does not work. This reason caused the low flow rate difference of the four prototypes. Subsequently, as the driving frequency continues to increase, the flow rate of the prototype with two elastic chambers is substantially higher than that of the other three prototypes. Besides, the flow rate of the prototype without elastic chamber is the lowest among the four prototypes. As indicated from the mentioned results, the elastic chamber works and decreases the dynamic load. The prototype with two elastic chambers not only improves self-priming performance, but also improves discharge performance, so its flow output is the best among the four prototypes. For the prototype without elastic chamber, the dynamic load weakens its self-priming performance, as well as its discharge performance, thus its output flow is the lowest among the four prototypes. Notably, the flow rate of the prototype with inlet elastic chamber is almost identical to that of the prototype with outlet elastic chamber. This is because the power loss caused by the dynamic load is the same for the two prototypes. Since the parameters of inlet and outlet pipelines are the same, the dynamic loads in the inlet and outlet pipelines are identical. Therefore, the power loss caused by the dynamic load in the prototype with inlet elastic chamber equals to that in the prototype with outlet elastic chamber. In totally, the above test results are basically consistent with the theoretical analysis in Section 2.

**Figure 9:**
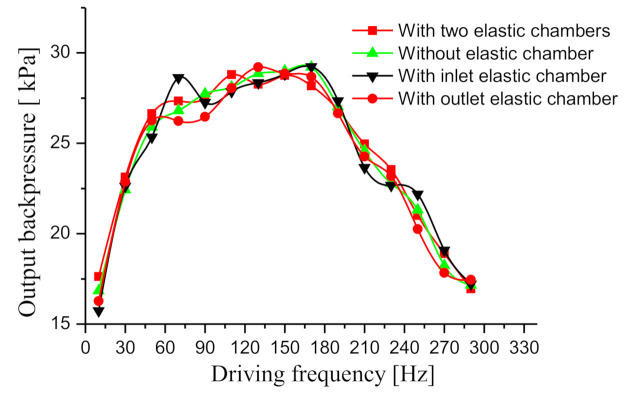
Backpressure curves of the four prototypes.

After the needle valve was closed, the maximum output backpressures of the four prototypes were tested. The test result is illustrated in Figure 9. As indicated from the test result, the elastic chamber does not help improve the maximum output backpressure. The flow rate of the four tested prototypes was zero, so there was almost no dynamic load in the pipelines. For this reason, the maximum output back pressure of the four prototypes is almost the identical.

### 4.2. Effect of the Elastic Diaphragm Stiffness on Flow Rate

**Figure 10:**
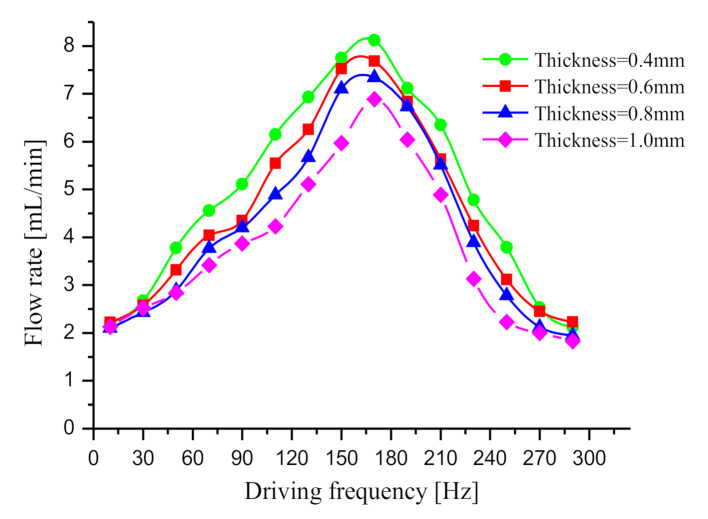
Flow rate curves of the prototypes with different thicknesses of the elastic diaphragm.

The stiffness of the elastic diaphragm in the elastic chamber has a critical effect on reducing the dynamic load. Since the thickness of elastic diaphragm directly determines its stiffness, the thickness of elastic diaphragm is used to represent its stiffness in the study. The smaller the thickness of elastic diaphragm, the smaller its rigidity. The thickness of elastic diaphragm was taken as 0.4, 0.6, 0.8 and 1 mm, respectively, to make four prototypes with two elastic chambers to perform flow tests. The other structural parameters of the four prototypes are the same as those of the prototype with two elastic chambers in Section 4.1. The flow rate test method and the driving voltage are the same as those in Section 4.1. The test result is given in Figure 10. As revealed from the test result, the flow rate increases with the decrease in the thickness of elastic diaphragm. Based on the analysis of Equations (11), (19), and (22) in Section 2, the flow rate curves are analyzed and explained. In the Equations, *k_f_* represents the stiffness, so the thinner the elastic diaphragm, the smaller the value of *k_f_*. According to Equation (11), decreasing *k_f2_* will decrease the amplitude amplification factor *H_2_*. It can be seen from Equations (19) and (22) that the smaller *k_f_* (*k_f1_* and *k_f2_*) and *H* (*H_1_* and *H_2_*), the smaller the dynamic load. Accordingly, adopting an elastic diaphragm with smaller stiffness is helpful to lower the dynamic load and increase the flow rate of the pump.

### 4.3. Effect of the Elastic Diaphragm Diameter on Flow Rate

**Figure 11:**
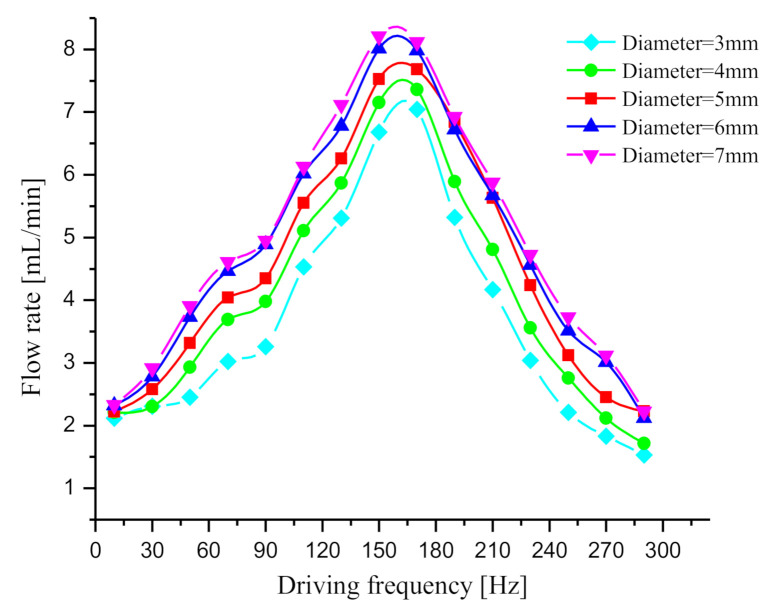
Flow rate curves of the prototypes with different diaphragm diameters.

The diameter of elastic diaphragm also acts as a critical parameter of elastic chamber. The diaphragm diameter was taken as 3, 4, 5, 6 and 7 mm, respectively, to make five prototypes with two elastic chambers to carry out flow test. The other structural parameters of the five prototypes are the same as those in Section 4.1. The flow rate test method and driving voltage are also the same as those in Section 4.1. The test result is illustrated in Figure 11. When the diaphragm diameter increased from 3 mm to 6 mm, the flow rate of prototype was obviously increased. However, the flow rate was not significantly increased when the diaphragm diameter increased from 6 mm to 7 mm. The main reason for this phenomenon is that the diaphragm diameter affects the elastic element *k_f_* and the damping element *c_f_* of the elastic chamber. On the one hand, the stiffness of the elastic diaphragm decreases with the diaphragm diameter. According to the analysis in Section 4.1, reducing the elastic stiffness *k_f_* will reduce the dynamic load, thereby increasing the pump flow, so increasing the diaphragm diameter can increase the flow rate of the pump. On the other hand, the volume of the elastic chamber and the liquid mass in the elastic chamber increases with the diaphragm diameter. According to the definition of dynamic load in Section 2, the larger the liquid mass, the greater the dynamic load. Based on the theory of fluid mechanics, the greater the liquid mass in the elastic chamber, the greater the head loss in the elastic chamber. In addition, the larger the diameter of the elastic diaphragm, the greater the energy dissipation caused by reciprocating motion. These factors are represented by the damping element *c_f_*, so increasing the diameter will increase the damping element *c_f_* and weaken the output flow of the pump. When the diameter is less than 6 mm, the weakening effect of damping element *c_f_* on the pump flow is relatively weak. However, the weakening effect is further enhanced when the diameter increases from 6 to 7, so the flow rate of the pump is not further improved.

As suggested from the test result, increasing the elastic diaphragm diameter within a certain range could improve the function of the elastic chamber, which helps lower the dynamic load of the liquid in the pipelines. Thus, adopting an elastic diaphragm with appropriate diameter could promote the output flow of the piezoelectric pump.

### 4.4. Effect of the Length of the Inlet and Outlet Pipelines on Flow Rate

**Figure 12:**
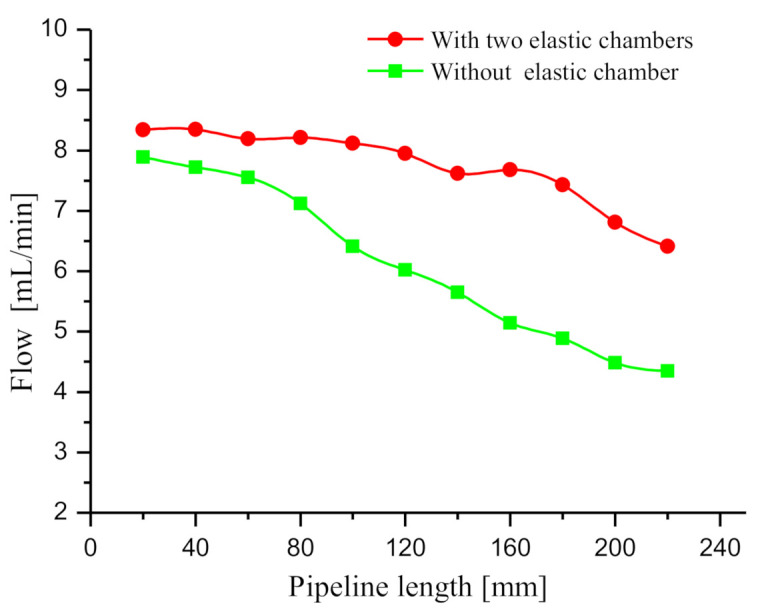
Relationship between flow rate and pipeline length.

The increase of the length of the inlet and outlet pipelines indicates the increase of the dynamic load of the liquid in the pipelines. To analyze the effect of pipeline length on the flow rate, the inlet and outlet pipelines were prepared with the lengths of 20, 40, 60, 80, 100, 120, 140, 160, 180, 200 and 220 mm. Subsequently, the flow rate of the prototypes with and without the elastic chambers was tested at different pipeline lengths. The elastic chamber diameter is 5 mm. The flow rate test method and driving voltage are the same as those mentioned in Section 4.1. The maximal flow rates of the tested prototypes with different pipeline lengths were recorded (Figure 12). The flow rate curves of the two prototypes decrease with the pipeline length, whereas the flow reduction rate of the prototype with two elastic chambers is significantly lower than that without elastic chamber. Based on Equation (11), we can analyze this phenomenon. The longer the pipeline, the greater the liquid mass in the pipeline will be. The increase of liquid mass leads to the noticeable increase of the dynamic load of liquid in the pipeline, so the increase of pipeline length decreases the output flow of the two prototypes. Since the elastic chamber can effectively decrease the dynamic load, the prototype with two elastic chambers has a significantly higher flow rate than the prototype without elastic chamber.

As indicated from the test result, long pipeline adversely affected the flow output of piezoelectric pumps as impacted the large dynamic load in the pipeline. Compared with conventional piezoelectric pump, piezoelectric pump with two elastic chambers achieves better flow output, and it is more suitable for long pipeline transmission system.

### 4.5. Effect of the Pump Chamber Height on the Output Performance

**Figure 13:**
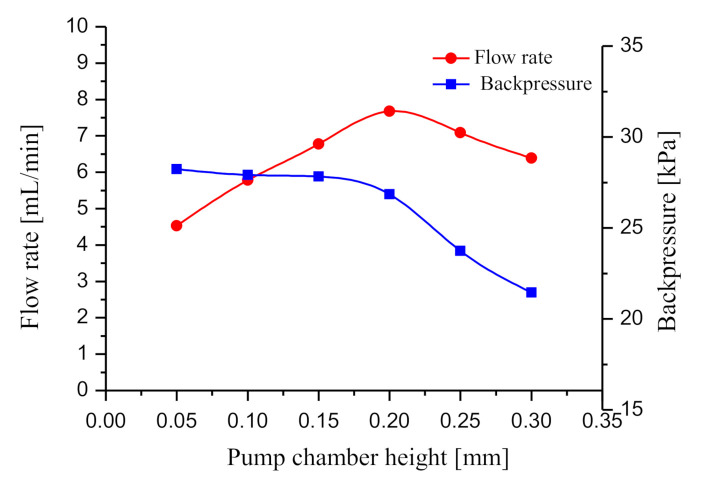
Output performance at different pump chamber heights.

Pump chamber height is a critical parameter for piezoelectric pumps. If the pump chamber is too high, it will down-regulate the fluid compression ratio; if it is too low, the flow resistance of the pump will increase. Accordingly, six prototypes were developed with different chamber heights (namely, 0.05, 0.1, 0.15, 0.2, 0.25 and 0.3 mm) to conduct tests. The other structural parameters of the six prototypes are the same as those of the prototype with two elastic chambers in Section 4.1. The driving voltage is 150 Vpp, and the driving frequency is 170 Hz. The output performance test results are presented in Figure 13. As revealed from the test result, as the chamber height increases, the flow rate curve increases first and then declines. At the height of 0.2 mm, the pump’s flow rate reaches the peak of 7.7 mL/min. At the height of 0.05 mm, the backpressure reaches the peak of 28.2 kPa. Differently, the output backpressure decreases with the chamber height. Based on Equations (23) and (24), the test result is analyzed. Reducing the height of the pump chamber helps to increase the pressure in the pump chamber. The increase of pressure in the pump chamber will increase the output flow and back pressure. However, the pressure loss in the pump chamber is inversely proportional to the square of the pump chamber height. When the height of pump chamber is too low (*h* < 0.2 mm), the pressure loss is very large. Therefore, a too low a pump chamber height will seriously reduce the output flow of the piezoelectric pump.

### 4.6. Experiments on Amplitude–Frequency Characteristics

**Figure 14:**
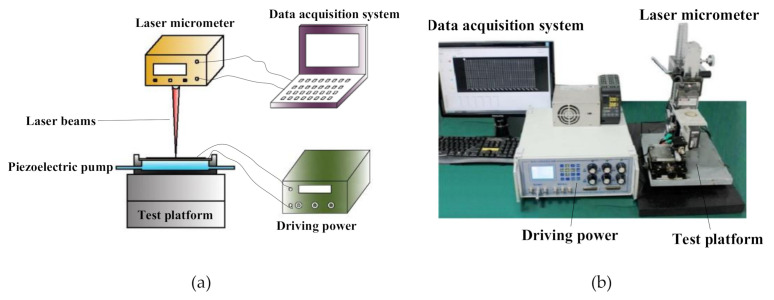
Amplitude measurement system of piezoelectric vibrator. (**a**) Schematic diagram of the amplitude measurement system; (**b**) photograph of the amplitude measurement system.

**Figure 15:**
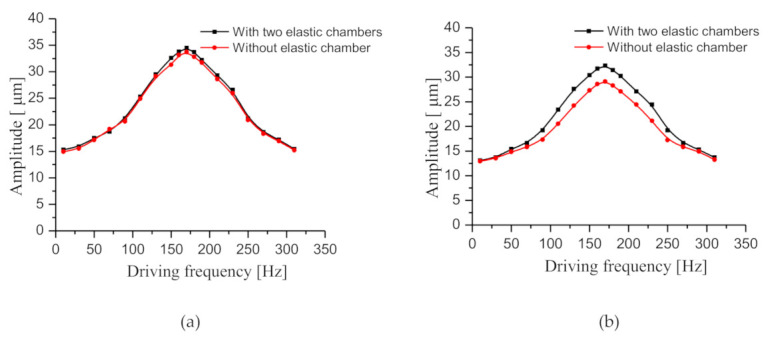
Amplitude–frequency characteristics of the prototypes with and without the elastic chambers. (**a**) The amplitude–frequency characteristics were tested without water pumping, (**b**) the amplitude–frequency characteristics were tested with water pumping.

The amplitude-frequency characteristics of the prototype with two elastic chambers and the prototype without elastic chamber were studied, respectively. The amplitude measurement system is shown in Figure 14. First, the amplitude-frequency characteristics of the two prototypes were tested without water pumping. After setting the peak-to-peak voltage of the driving power as 150 V, we changed the driving frequency and measured the amplitude of piezoelectric vibrator by laser micrometer. The test result is shown in Figure 15a, as the driving frequency increases, the vibrator amplitudes of the two prototypes both initially increase, but then declines. There is almost no difference between the amplitudes of the two prototypes. When the driving frequency is 170 Hz, the vibrator amplitudes of the two prototypes reach the maximum values of 34.5 μm and 33.7 μm, respectively. Then the amplitude-frequency characteristics of the two prototypes were tested with water pumping, using the same test method as above. The test result is shown in Figure 15b. Obviously, the trend of amplitude curves in Figure 15a,b is similar, but the amplitudes in Figure 15b are both slightly smaller than those in Figure 15a. Notably, Figure 15b shows that the amplitude of the prototype with two elastic chambers is higher than that of the prototype without elastic chamber. As the driving frequency increases, the amplitude difference between the two prototypes initially increases, but then declines. When the driving frequency is 170 Hz, the amplitudes reach the maximum values of 32.3 μm and 29.1 μm, respectively. The maximum amplitude difference is 3.2 μm.

When the prototypes pump water, the dynamic load and flow resistance of water in the pump system produce resistance to the piezoelectric vibrator, so the amplitude of the piezoelectric vibrator decreases slightly. The elastic chamber can effectively reduce the dynamic load, so the amplitude of the prototype with the two elastic chambers is higher than that of the prototype without the elastic chamber. According to the analysis in Section 2, the dynamic load is proportional to the amplitude of the piezoelectric vibrator and the flow rate. Therefore, as the driving frequency increases, the dynamic load initially increases, but then declines. As a result, the amplitude difference increases first and then decreases.

We can see from Figure 15 that the resonance frequency of the vibrator is about 170 Hz. In Section 4.1, we can see from Figure 8 that the optimal driving frequencies of the prototypes are all around 170 Hz. Therefore, we can infer that the elastic chamber does not have much influence on the resonance frequency of the prototype and the optimal drive frequency of the prototype is mainly determined by the vibrator structure. In our previous study [29], we performed a check valve optimization experiment. The stiffness of check valve is directly related to its resonance frequency. When the resonance frequency of the check valve is lower than the resonance frequency of the piezoelectric vibrator, two peaks will appear in the flow rate curve of the pump. However, in the present study, the system flow rate curves did not appear two peaks, so we infer that the resonance frequency of the check valve should be higher than that of the vibrator.

### 4.7. Effect of the Backpressure on the Flow Rate

**Figure 16:**
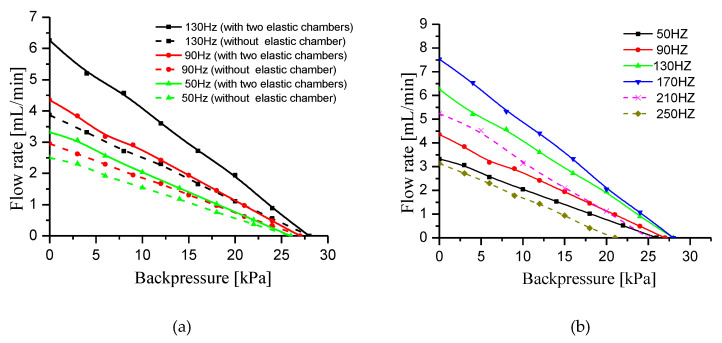
Relationship between flow rate and backpressure at different driving frequency. (**a**) Backpressure comparison curves of the prototype with two elastic chambers and the prototype without elastic chamber, (**b**) backpressure curves of the prototype with two elastic chambers.

Backpressure performance tests were conducted on the prototype with two elastic chambers and the prototype without elastic chamber. Figure 16a shows the relationship between flow rate and backpressure of the two prototypes at three driving frequencies, namely, 130, 90 and 50 Hz. Figure 16b shows the backpressure curves of the prototype with two elastic chambers at six driving frequencies, namely, 50, 90, 130, 170, 210 and 250 Hz. The driving voltage in the test (Figure 16) is 150 Vpp. Figure 16a,b show that the flow rate decreases approximately linearly with backpressure at each driving frequency. Notably, in Figure 16a, the flow rate difference between the two prototypes gradually decreases with the backpressure at each driving frequency. The reason for this phenomenon can be explained as follows. The flow rate in the pipelines decreases with the backpressure, so the dynamic load of the liquid in the pipelines decreases with the backpressure. The smaller the dynamic load, the smaller its effect on the flow rate. Therefore, the flow rate difference between the two prototypes decreases with the backpressure. In addition, it can be found from Figure 16b that the flow rate increases first and then decreases with the driving frequency at the same backpressure. This phenomenon is consistent with the change trend of flow rate in Section 4.1.

**Figure 17:**
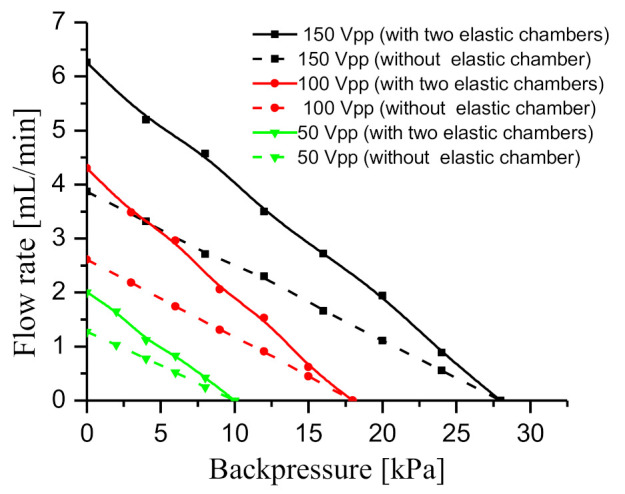
Relationship between flow rate and backpressure at different driving voltages.

Figure 17 shows the relationship between flow rate and backpressure of the two prototypes at three driving voltages, namely, 50, 100 and 150 Vpp. Obviously, the test result shows that the flow rate decreases approximately linearly with backpressure at each driving voltage. Similar to Figure 16a, the flow rate difference between the two prototypes gradually decreases with the backpressure at each driving voltage. The reason for this phenomenon is similar to the analysis in Figure 16a. In addition, as the voltage increases, the flow rate of the two prototypes increases at the same backpressure. The vibration displacement of piezoelectric vibrator increases with the driving voltage, so the flow rate increases with the voltage.

## 5. Conclusions

In this study, a piezoelectric pump with two elastic chambers was investigated, in which the inlet and outlet elastic chambers were used to decrease the dynamic load of the liquid in the inlet and outlet pipelines. We have established a mathematical model of the piezoelectric pump with two elastic chambers and theoretically analyzed the performance of the pump and the effect of different structural parameters on its performance. Subsequently, prototypes with different structural parameters were developed and tested. Experimental results are presented to analyze the effect of several key parameters on the output performance of the prototype with two elastic chambers, which helps optimize the pump system.

The elastic chamber decreased the dynamic load of the liquid in the pipelines and improved the flow rate of the prototype. The flow rate of the prototype with two elastic chambers was higher than that of the prototype with one elastic chamber and the prototype without an elastic chamber. The maximal flow rate of the prototype with two elastic chambers was nearly 36% higher than that of the prototype without elastic chamber, however, the elastic chamber did not increase the output backpressure of the pump.

Adopting an elastic diaphragm with smaller stiffness or larger diameter was beneficial to decrease the dynamic load of the liquid and increase the flow rate of the pump. As the length of the inlet and outlet pipelines increased, the dynamic load of the liquid in the pipelines gradually increased, so the output flow rate of the prototype gradually decreased. Long pipelines adversely affected the flow output of piezoelectric pumps under large dynamic load in the pipelines. Compared with conventional piezoelectric pump, the piezoelectric pump with two elastic chambers had better flow output and was more suitable for long pipeline transmission system.

The pump chamber height had an important effect on the output performance of the prototype with two elastic chambers. As the chamber height increased, the flow rate increased first and then declined, but the output backpressure always decreased. At the height of 0.2 mm, the flow rate was peaked at 7.7 mL/min; at the height of 0.05 mm, the output backpressure reached the maximum of 28.2 kPa.

The dynamic load of the liquid in the pipelines produced resistance to the piezoelectric vibrator, reducing the amplitude of the piezoelectric vibrator to a certain extent. However, the prototype with two elastic chambers could effectively reduce the adverse impact of dynamic load on the piezoelectric vibrator. The flow rate decreased almost linearly with the backpressure. The flow rate of the prototype with two elastic chambers was higher than that of the prototype without elastic chamber at the same backpressure, and the flow rate difference between the two prototypes gradually decreased with the backpressure.

## Figures and Tables

**Table 1:** The main structural parameters of the prototype with two elastic chambers.

Structural Parameters	Values
$\mathrm{Diameter}\;\mathrm{of}\;\mathrm{the}\;\mathrm{piezoelectric}\;\mathrm{vibrator}\;D_{v}\;(\mathrm{mm})$	20
$\mathrm{Diameter}\;\mathrm{of}\;\mathrm{the}\;\mathrm{pump}\;\mathrm{chamber}\;D_{p}\;(\mathrm{mm})$	16
Diameter of the fixed part of the valve piece *d_s_* (mm)	7
Diameter of the moving part of the valve piece *d_m_* (mm)	1.4
Thickness of the valve piece (mm)	0.03
Thickness of the valve plate (mm)	0.05
Stiffness of the wheeled check valve *k_v_* (N/m)	355.31
$\mathrm{Height}\;\mathrm{of}\;\mathrm{the}\;\mathrm{pump}\;\mathrm{chamber}\;H_{p}\;(\mathrm{mm})$	0.05, 0.1, 0.15, 0.2, 0.25, 0.3
Diameter of inlet and outlet flow channel *D_f_* (mm)	1.5
$\mathrm{Thickness}\;\mathrm{of}\;\mathrm{the}\;\mathrm{elastic}\;\mathrm{diaphragm}\;T_{e}\;(\mathrm{mm})$	0.4, 0.6, 0.8, 1
Height of the elastic chamber *H_e_* (mm)	2
Diameter of the elastic diaphragm *D_e_* (mm)	3,4,5,6,7
